# Artificial intelligence in dysphagia since the 21st century: a bibliometric and visualization study

**DOI:** 10.3389/fmed.2025.1624381

**Published:** 2025-08-18

**Authors:** Tao Liu, Yuetong Rong, Dan Li, Heli Zhang, Baohua Li, Guoqing Cui, Shaomei Shang

**Affiliations:** ^1^Peking University School of Nursing, Beijing, China; ^2^Department of Nursing, Peking University Third Hospital, Beijing, China; ^3^Department of Rehabilitation, Peking University Third Hospital, Beijing, China

**Keywords:** dysphagia, swallowing, artificial intelligence, bibliometric, VOSviewer

## Abstract

**Background:**

The fields of dysphagia is progressively acknowledging the transformative capacity of artificial intelligence (AI). The implementation of this technology is profoundly impacting research directions, clinical practices, and healthcare systems. However, existing studies remain scattered and predominantly focus on specific techniques or case applications, lacking a systematic synthesis of global research output, influential contributors, collaboration networks, and evolving thematic trends. A comprehensive bibliometric review is therefore essential to map the current landscape and guide future interdisciplinary research.

**Methods:**

This study applies bibliometric and visual analysis methods to comprehensively review the global research activities in AI in dysphagia. Data from 633 articles published by 3,533 authors in 292 journals from January 2000 to February 2025 in Web of Science Core Collection (WoSCC) database were collected and analyzed to identify top publications, sources, authors, institutions, countries/regions, and keywords.

**Results:**

The research activity of AI in dysphagia, which shows an overall upward trend that can be divided into three distinct periods: the first phase (2000–2012), the second phase (2013–2017) and the third phase (2018-Present). The most cited article was Radiotherapy vs. transoral robotic surgery and neck dissection for oropharyngeal squamous cell carcinoma (ORATOR): an open-label, phase 2, randomized trial (344 citations). The most prolific journal was Head and Neck—Journal for the Sciences and Specialties of the Head and Neck with 30 publications. Sejdic Ervin (28 articles), the University of Pittsburgh (39 articles), and the USA (255 articles) were the leading author, institution, and country, respectively. Dysphagia was the most frequently occurring keyword (286 occurrences), while emerging terms included machine learning (ML) and deep learning (DL).

**Conclusion:**

This bibliometric analysis reveals the evolving landscape of AI research in dysphagia, highlighting current hotspots and future directions. AI is driving significant shifts in both research and clinical practice in dysphagia; however, challenges such as interdisciplinary integration and ethical considerations remain to be addressed.

## 1 Introduction

Dysphagia is a clinical symptom of swallowing dysfunction, impairing the safe and transport of solids and/or liquids from the oral cavity to the stomach (1). It affects 13.4% of the global population (2), with prevalence exceeding 40% in specific patient populations including stroke, sarcopenia, parkinson's disease, dementia, and geriatric patients (3). Anatomically, dysphagia is typically categorized into oropharyngeal dysphagia (OD) and esophageal dysphagia (ED) (1). OD is often attributed to neurologic diseases or head and neck malignancies, and is clinically manifested by coughing, choking, nasal regurgitation and aspiration pneumonia (4). ED commonly resulting from motility disorders, strictures or esophageal tumors, presents a sensation of food sticking in the throat or chest (5). In addition to dysphagia, including dryness, silent aspiration, protein-energy malnutrition and recurrent pulmonary infections (3), recent epidemiologic data reveal a significant increase in adverse outcomes: the intubation rate in the cohort was more than twice as high in patients with dysphagia (34%) compared to patients without dysphagia (6). There were also longer length of hospital stay and mechanical ventilation in the dysphagic patients, which may be attributed to factors such as inadequate oral intake, aspiration pneumonia, and secondary infections (7).

Current diagnostic paradigms rely heavily on a range of instrumental assessments, including videofluoroscopic swallow studies (VFSS), fiberoptic endoscopic evaluation of swallowing (FEES), high-resolution manometry (HRM), electromyography (EMG), electrokinesiographic study of swallowing (EKSS), and computed tomography (CT) (8). Among these, VFSS and FEES are widely regarded as the gold standards, providing dynamic visualization of pharyngeal phase physiology and objective quantification of penetration-aspiration events (9). However, their widespread application is often limited by the availability of equipment and trained personnel, particularly in resource-constrained settings, leading to geographical and socioeconomic disparities in care (10).

The treatment of dysphagia is highly individualized, depending on the etiology, anatomical site and severity. Current therapeutic approaches generally fall into four categories: compensatory, facilitative, rehabilitative and restorative techniques (11). Compensatory strategies such as postural adjustments such chin-tuck or head rotation, and dietary or bolus modification, aim to reduce aspiration risk without altering swallowing physiology (12, 13). Facilitation approaches, including thermal tactile stimulation (TTS) and sour bolus, are used to enhance reflexive swallowing response (14, 15). Rehabilitative methods emphasize neuromuscular retraining through targeted exercises such as the Mendelsohn maneuver and Shaker exercise (16). Restorative techniques, such as neuromuscular electrical stimulation, biofeedback-assisted therapy, or task-specific swallowing training, seek to improve biomechanical coordination and promote long-term recovery (17, 18). In severe cases, when dysphagia is so severe that the nutritional demands cannot be covered orally, artificial nutrition has to be considered (16).

In recent years, artificial intelligence (AI) has begun to reshape the dysphagia research landscape through three transformative pathways: (1) automated interpretation of multidimensional clinical metrics (19), (2) predictive modeling of therapeutic outcomes (20), and (3) improving clinical decision support (21). Lee et al. (22) used a deep learning (DL) model to detect airway invasion from VFSS images, without clinician input, with 97.2% accuracy in classifying image frames and 93.2% in classifying video files. Beyond traditional computer vision applications, Li et al. (23) used multi-layer perceptron (MLP), convolutional neural network (CNN), and convolutional recurrent neural network (CRNN) models to classify and identify swallowing-related activities based on their acoustic signatures, achieving classification accuracies of 74% (MLP), 68% (CNN), and 54% (CRNN), respectively. These technologies not only promise improved diagnostic accuracy and early detection, but also open new avenues for real-time monitoring and individualized therapy. However, the integration of AI into dysphagia care is still at an early stage, and progress is uneven across disciplines and geographic regions.

To date, there has been no systematic, data-driven evaluation of how AI is being deployed in the study and management of dysphagia. While interest in this domain is growing, the lack of a comprehensive overview makes it difficult to identify leading contributors, technological trajectories, and emergent research priorities. Bibliometric and science mapping approaches offer a powerful lens through which to assess the structure and dynamics of this interdisciplinary field, revealing both the strengths and gaps in current knowledge (24).

In this study, we conduct the first global bibliometric and visualization analysis of research on AI in dysphagia, covering a 25-year span from 2000 to early 2025. Using tools such as VOSviewer and the Bibliometrix package in R, we quantitatively map the evolution of publications, authorship networks, institutional and national contributions, and thematic clusters of research. By doing so, we aim to (1) characterize the structure and dynamics of the existing research landscape, (2) identify dominant and emerging themes in the integration of AI with dysphagia, and (3) inform future directions at the intersection of digital health, clinical neuroscience, and rehabilitation medicine.

## 2 Methods

### 2.1 Data source

The Web of Science Core Collection (WoSCC) was selected as the primary data source for this bibliometric analysis. WoSCC, maintained by Clarivate Analytics, is widely recognized as one of the most authoritative and commonly used databases for academic literature retrieval and citation analysis (25). The dataset used in this study is publicly available on the Open Science Framework (OSF) platform (doi: 10.17605/OSF.IO/S3HNW).

### 2.2 Search strategies

**Figure 1:**
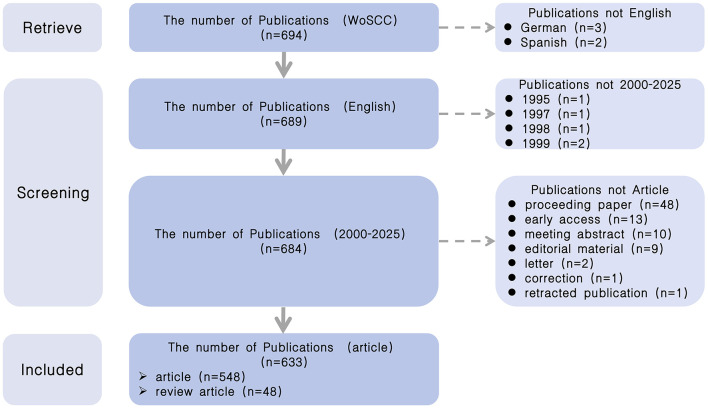
Publication selection flow diagram. This diagram illustrates the screening process and final inclusion of publications related to AI in dysphagia.

This study defined appropriate keywords for the search after reviewing related literature on AI and dysphagia research (26, 27). All data were retrieved on February 10, 2025 from WoSCC with the following strategies: (1) TS = (“artificial intelligence” OR “robotic^*^” OR “expert^*^ system^*^” OR “intelligent learning” OR “feature^*^ extraction” OR “feature^*^ mining” OR “feature^*^ learning” OR “machine learning” OR “feature^*^ selection” OR “unsupervised clustering” OR “image^*^ segmentation” OR “supervised learning” OR “semantic segmentation” OR “deep network^*^” OR “Bayes^*^ network” OR “deep learning” OR “neural network^*^” OR “neural learning” OR “neural nets model” OR “artificial neural network” OR “data mining” OR “graph mining” OR “data clustering” OR “big data” OR “knowledge graph”) AND (“dysphagia” OR “swallowing disorder” OR “swallowing difficulty” OR “swallowing impairment” OR “swallowing dysfunction” OR “swallowing problem” OR “aphagia” OR “deglutition difficulty” OR “ingurgitation difficulty”); (2) Language = English; (3) Timespan = 2000–2025; (4) Document Type = article and review article. Full records and cited references are exported and downloaded in plain text format for analysis. The retrieval, screening, and enrollment process is shown in Figure 1.

### 2.3 Analysis tools

Microsoft Excel is a widely accessible data analysis and visualization tool, commonly provided by institutions for research and administrative purposes (28). Excel (version 16.90) was employed to analyze the annual publication volume and trends, as well as to generate corresponding line charts.

VOSviewer (version 1.6.20), developed by Nees Jan van Eck and Ludo Waltman at Leiden University in 2010, is a freely available software tool widely used for constructing and visualizing bibliometric networks (29). In this study, VOSviewer was utilized to analyze and visualize co-authorship, co-occurrence, and bibliographic coupling relationships across publications, journals, authors, countries, institutions, and keywords.

Bibliometrix, an open-source R package developed by Aria and Cuccurullo (30), was used for advanced bibliometric analysis and science mapping. R (version 2024.12.11) was specifically applied to visualize the Three-Field Plot, international collaboration networks, and the scientific output of countries and affiliations.

## 3 Results

### 3.1 Analysis of publications

**Figure 2:**
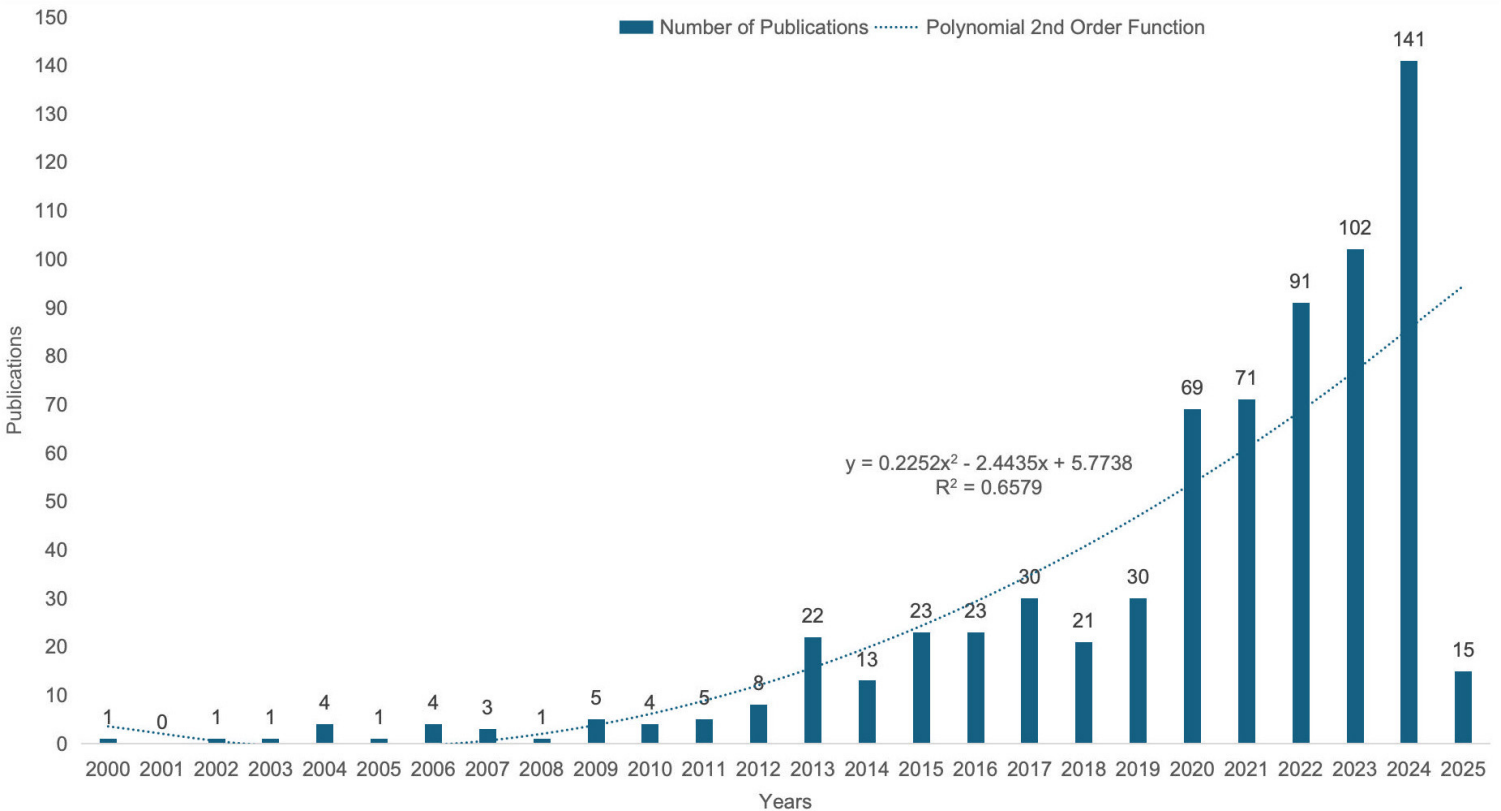
The Annual Number and Trend of Publications of AI in Dysphagia (2000–2025). The number of publications shows an overall increasing trend, with a marked acceleration after 2018, reflecting growing research interest in this field.

From 2000 to 2025, the number of publications on AI in dysphagia research exhibited an overall upward trajectory, which can be divided into three distinct phases (Figure 2). The first phase (2000–2012) was marked by a limited number of publications, reflecting the nascent stage of research in this domain. The second phase (2013–2017) showed irregular fluctuations in publication volume, indicating a period of exploration and gradual development. The third phase (2018–present) has been characterized by a sustained and significant increase in the number of articles, suggesting growing interest and advancements in this field.

A total of 633 articles have been published, with an average annual growth rate of 13.6%, reflecting the accelerating pace of research in this area. Among these, 16 publications have received more than 100 citations. Table 1 lists the top 10 most-cited articles, led by the study “Radiotherapy vs. transoral robotic surgery and neck dissection for oropharyngeal squamous cell carcinoma (ORATOR): an open-label, phase 2, randomized trial” (31), published in 2019, which has accrued 369 citations.

**Table 1:** The top 10 cited publications of AI in dysphagia.

**Ranking**	**Title**	**Author (Year)**	**DOI**	**IF**	**Citation**
1	Radiotherapy versus transoral robotic surgery and neck dissection for oropharyngeal squamous cell carcinoma (ORATOR): an open-label, phase 2, randomized trial	Nichols et al. (2019)	10.1016/S1470-2045(19)30410-3	41.6	369
2	Phase II Randomized Trial of Transoral Surgery and Low-Dose Intensity Modulated Radiation Therapy in Resectable p16+Locally Advanced Oropharynx Cancer: An ECOG-ACRIN Cancer Research Group Trial (E3311)	Ferris et al. (2022)	10.1200/JCO.21.01752	42.1	267
3	Functional outcomes after transoral robotic surgery for head and neck cancer	Iseli et al. (2009)	10.1016/j.otohns.2009.05.014	2.6	190
4	Functional outcomes after TORS for oropharyngeal cancer: a systematic review	Hutcheson et al. (2014)	10.1007/s00405-014-2985-7	1.9	163
5	A new paradigm for the diagnosis and management of unknown primary tumors of the head and neck: A role for transoral robotic surgery	Mehta et al. (2013)	10.1002/lary.23562	2.2	136
6	Quality of life in survivors of oropharyngeal cancer: A systematic review and meta-analysis of 1,366 patients	Roets et al. (2018)	10.1016/j.ejca.2017.03.006	2.8	132
7	Repetitive transcranial magnetic stimulation in stroke rehabilitation: review of the current evidence and pitfalls	Fisicaro et al. (2019)	10.1177/1756286419878317	4.7	127
8	Cardiovascular causes of airway compression	Kussman et al. (2004)	10.1046/j.1460-9592.2003.01192.x	1.7	127
9	Transoral Endoscopic Head and Neck Surgery and Its Role Within the Multidisciplinary Treatment Paradigm of Oropharynx Cancer: Robotics, Lasers, and Clinical Trials	Holsinger et al. (2015)	10.1200/JCO.2015.62.3157	42.1	132
10	Functional Swallowing Outcomes Following Transoral Robotic Surgery vs Primary Chemoradiotherapy in Patients With Advanced-Stage Oropharynx and Supraglottis Cancers	More et al. (2013)	10.1001/jamaoto.2013.1074	6.0	117

### 3.2 Analysis of sources

A total of 292 journals have published articles related to the application of artificial intelligence in dysphagia. Table 2 presents the top 10 journals ranked by the number of publications, among which 8 journals have published at least 10 articles. Notably, Head and Neck—Journal for the Sciences and Specialties of the Head and Neck ranks first, with a total of 30 (4.74%) documents in this field.

**Table 2:** The top 10 journals of AI in dysphagia.

**Ranking**	**Journal**	**Country**	**JCR**	**IF**	**Documents**	**Total link strength**
1	Head and Neck-Journal for the Sciences and Specialties of the Head and Neck	USA	Q1	2.4	30 (4.74%)	494.06
2	Surgical Endoscopy and Other Interventional Techniques	USA	Q1	2.4	21 (3.32%)	121.28
3	Dysphagia	USA	Q2	2.2	19 (3.00%)	181.9
4	Laryngoscope	USA	Q2	2.2	17 (2.69%)	192.27
5	European Archives of Oto-Rhino-Laryngology	Germany	Q2	1.9	14 (2.21%)	290.69
6	Oral Oncology	UK	Q1	4.0	14 (2.21%)	204.37
7	Scientific Reports	UK	Q2	3.8	13 (2.05%)	127.18
8	JAMA Otolaryngology-Head & Neck Surgery	USA	Q1	6.1	11 (1.74%)	202.76
9	Auris Nasus Larynx	Netherlands	Q3	1.6	8 (1.26%)	123.29
10	Frontiers in Neurology	Switzerland	Q2	2.7	7 (1.11%)	154.04

### 3.3 Analysis of authors

**Figure 3:**
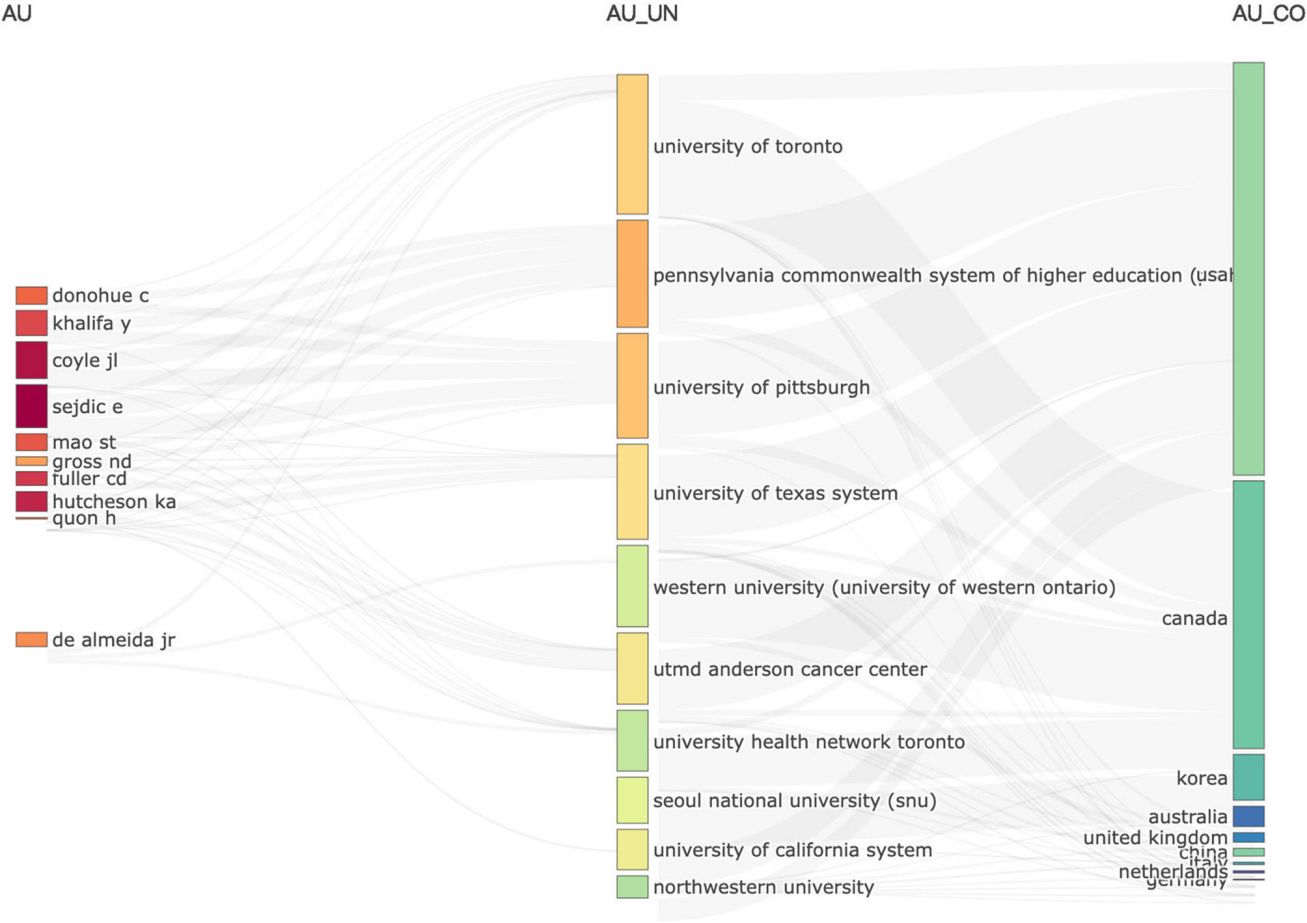
Three-field plot visualized using bibliometrix. This bibliometric map displays the interrelationships among the most productive authors **(left)**, institutions (center), and countries **(right)**. Thicker lines and larger nodes indicate stronger collaboration and higher publication volume.

A total of 3,533 authors have contributed to research articles on artificial intelligence in dysphagia, and the institutions and countries of high contributing authors are visualized in Figure 3. Table 3 lists the top 10 most productive authors in this domain, among whom 5 have published at least 10 articles. Notably, Ervin Sejdić emerges as the most prolific contributor, with a total of 28 (4.42%) documents, highlighting his prominent role in advancing research in this area.

**Table 3:** The top 10 productive authors of AI in Dysphagia.

**Ranking**	**Author**	**Organization**	**Country**	**Subject categories**	**Documents (%)**	**Total link strength**
1	Sejdic Ervin (28)	University of Toronto	Canada	Engineering, Computer Science, Neurosciences & Neurology, Science & Technology, Medical Informatics	28 (4.42%)	28.00
2	Coyle James L. (21)	University of Pittsburgh	USA	Engineering, Computer Science, Otorhinolaryngology, Rehabilitation, Neurosciences & Neurology	21 (3.32%)	21.00
3	Khalifa Yassin (13)	University of Pittsburgh	USA	Engineering, Computer Science, Cardiovascular System & Cardiology, Otorhinolaryngology, Science & Technology	12 (2.05%)	13.00
4	Hutcheson Katherine A. (11)	UTMD Anderson Cancer Center	USA	Oncology, Radiology, Nuclear Medicine & Medical Imaging, Otorhinolaryngology, Surgery, Research & Experimental Medicine	11 (1.74%)	9.00
5	Mao Shitong (10)	UTMD Anderson Cancer Center	USA	Engineering, Computer Science, Otorhinolaryngology, Science & Technology, Materials Science	10 (1.58%)	10.00
6	Donohue Cara (9)	Vanderbilt University Medical Center	USA	Neurosciences & Neurology, Rehabilitation, Linguistics, Audiology & Speech-Language Pathology, Otorhinolaryngology	9 (1.42%)	9.00
7	Clifton D. Fuller (7)	UTMD Anderson Cancer Center	USA	Oncology, Radiology, Nuclear Medicine & Medical Imaging, Otorhinolaryngology, Surgery, Science & Technology	7 (1.11%)	7.00
8	Chau Tom (7)	Holland Bloorview Kids Rehabilitation Hospital	Canada	Engineering, Neurosciences & Neurology, Rehabilitation, Computer Science, Sport Sciences	7 (1.11%)	6.00
9	Quon Harry (7)	Johns Hopkins University	USA	Oncology, Otorhinolaryngology, Radiology, Nuclear Medicine & Medical Imaging, Surgery, Research & Experimental Medicine	7 (1.11%)	6.00
10	Meccariello Giuseppe (6)	Azienda USL della Romagna	Italy	Otorhinolaryngology, Surgery, General & Internal Medicine, Oncology, Neurosciences & Neurology	6 (0.95%)	6.00

### 3.4 Analysis of institutions and countries

**Figure 4:**
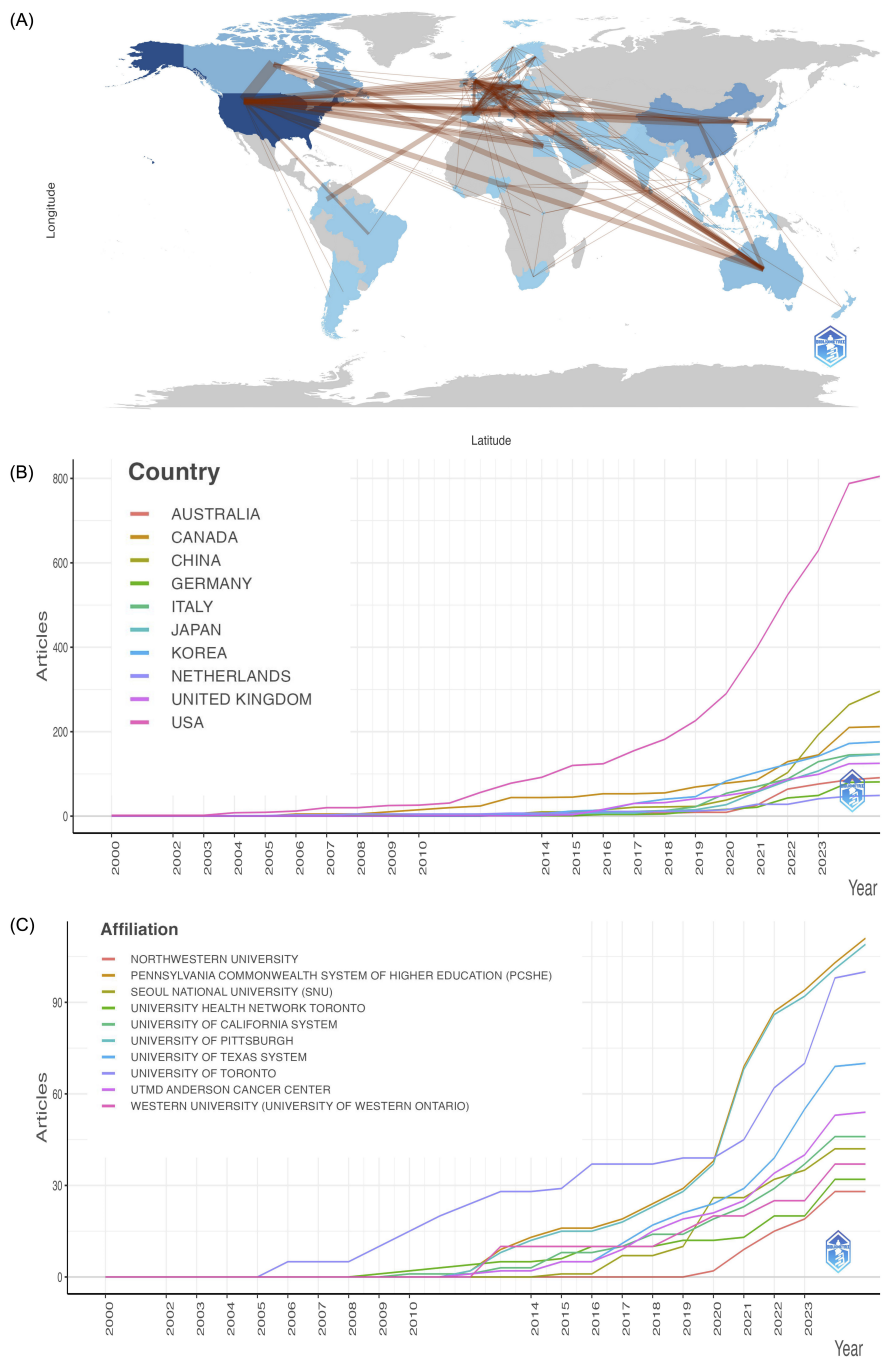
**(A)** Countries' collaboration world map visualized using bibliometrix. Deeper blues signal more extensive collaboration, with thicker lines indicating stronger collaborative ties. **(B)** Countries' production over time. **(C)** Affiliations' production over time.

A total of 1,055 institutions have contributed to research on artificial intelligence in dysphagia. As shown in Table 4, the top 10 most productive institutions are identified, among which 8 have published at least 10 articles. The University of Pittsburgh ranks first with 39 publications (6.16%), highlighting its leading influence in the field. Notably, the Pennsylvania Commonwealth System of Higher Education (PCSHE) has recently emerged as the most productive affiliation, with its increasing output over time further confirmed by the trends shown in Figure 4C.

**Table 4:** The top institutions and countries of AI in dysphagia.

**Ranking**	**Institution**	**Documents (%)**	**Total link strength**	**Country**	**Documents (%)**	**Total link strength**
1	University of Pittsburgh	39 (6.16%)	15.00	USA	255 (40.28%)	60.00
2	University of Toronto	31 (4.90%)	23.00	China	81 (12.80%)	13.00
3	UTMD Anderson Cancer Center	24 (3.79%)	15.00	Canada	51 (8.07%)	28.00
4	Soul National University	12 (1.90%)	3.00	Japan	50 (7.90%)	4.00
5	North York General Hospital	10 (1.58%)	10.00	South Korea	47 (7.42%)	6.00
6	Newcastle University	10 (1.58%)	8.00	England	46 (7.27%)	22.00
7	University of California San Diego	10 (1.58%)	3.00	Italy	39 (6.16%)	16.00
8	Northwestern University	10 (1.58%)	1.00	Australia	25 (3.95%)	15.00
9	Korea University	9 (1.42%)	4.00	Germany	21 (3.32%)	10.00
10	University of Pennsylvania	9 (1.42%)	4.00	Netherlands	17 (2.69%)	5.00

Research in this area spans across 65 countries, with 11 of them having published 10 or more articles. Table 4 and Figure 4B list the top 10 countries/regions by publication volume. The United States stands out as the most prolific contributor, accounting for 255 publications (40.28%), underscoring its dominant role in advancing AI-related dysphagia research globally. In addition, Figure 4A visualizes the global collaboration network, indicating that the lack of knowledge sharing and cooperation across countries and the USA demonstrated the strongest international collaboration.

### 3.5 Analysis of keywords

**Figure 5:**
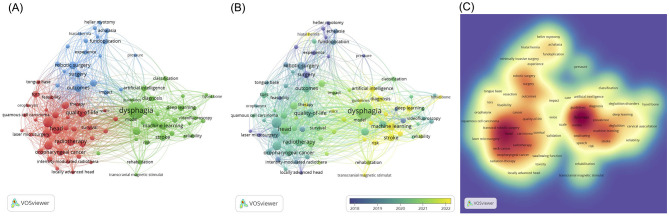
Keywords of AI in Dysphagia visualized using VOSviewer. **(A)** Knowledge map of keywords. Larger circles indicate higher frequency; thicker lines represent stronger connections; different colors correspond to distinct clusters. **(B)** Timeline of keywords. Keywords closer to yellow represent more recently appearing terms. **(C)** Hotpots of Keywords. Keywords closer to red indicate higher research intensity or popularity.

A keyword co-occurrence knowledge map related to AI in dysphagia is presented in Figure 5A. Among the 2,586 keywords identified, 14 appeared 50 times or more. These high-frequency keywords, listed in descending order of occurrence, include: dysphagia, head, transoral robotic surgery, radiotherapy, squamous-cell carcinoma, quality of life, neck cancer, outcomes, robotic surgery, swallowing, machine learning, aspiration, oropharyngeal cancer, and cancer. Table 5 displays the top 10 most frequently occurring keywords, with dysphagia ranking first, appearing 286 times.

**Table 5:** The top 10 keywords of AI in dysphagia.

**Ranking**	**Keywords**	**Occurrences (%)**	**Total link strength**
1	Dysphagia	286 (11.06%)	267.00
2	Head	99 (3.83%)	97.00
3	Transoral robotic surgery	96 ()3.71%	92.00
4	Radiotherapy	78 (3.02%)	75.00
5	Squamous-cell carcinoma	75 (2.90%)	75.00
6	Quality of life	75 (2.90%)	75.00
7	Neck cancer	72 (2.78%)	72.00
8	Outcomes	62 (2.40%)	61.00
9	Robotic surgery	58 (2.24%)	53.00
10	Swallowing	56 (2.17%)	54.00

As shown in Figure 5B, the primary research trends in AI and dysphagia have undergone substantial evolution over time. Early research predominantly emphasized surgical interventions, including robotic surgery, minimally invasive surgery, and Heller myotomy. In contrast, recent years have seen the emergence of three prominent thematic trends: (1) A growing focus on diagnostic and rehabilitative approaches, with increasing attention to voice, speech, clinical scales, and transcranial magnetic stimulation; (2) Deeper investigation into the etiology and complications of dysphagia, addressing aspects such as classification, risk factors, stroke, oropharyngeal dysphagia, and pneumonia; (3) Advancement of AI-based analytical techniques, particularly emphasizing machine learning (ML) and DL methodologies.

The current research hotspots in the field, as visualized in Figure 5C, cluster around three major thematic areas: (1) Etiology and complications of dysphagia, involving terms such as head, neck cancer, stroke, oropharyngeal cancer, carcinoma, swallowing, and speech. (2) Therapeutic strategies for dysphagia, including radiotherapy, transoral robotic surgery, and laser microsurgery. (3) AI models and algorithms, with a particular focus on machine learning, deep learning, and intelligent modeling approaches.

## 4 Discussion

This study analysis highlights the dynamic evolution of global research on AI in dysphagia since the beginning of the 21st century. The developmental trajectory can be broadly categorized into three phases: a period of slow growth (2000–2012), a stage of fluctuating progress (2013–2017), and a phase of exponential expansion (2018–present). These stages reflect both the opportunities and limitations inherent in applying AI technologies to the complex clinical landscape of dysphagia. Notably, the steep rise in publications after 2018 coincides with significant advancements in ML and deep DL, as well as their expanding applications in biomedical research domains (32–34). The United States has emerged as the most productive country, contributing 255 publications, underscoring its leadership in this interdisciplinary field. However, the relatively fragmented global collaboration network suggests a critical gap in cross-national knowledge sharing and cooperative development.

## 5 Shifting study paradigm

The keyword co-occurrence and temporal analysis reveal a marked paradigm shift in the research landscape—from early investigations centered on surgical techniques such as transoral robotic surgery to AI-driven approaches in diagnosis, therapy, and rehabilitation. For example, a landmark article with 190 citations reported on functional outcomes following transoral robotic surgery for head and neck cancer, reflecting the emphasis on surgical innovation in earlier years (35). More recent studies, however, have increasingly focused on non-invasive, technology-assisted methods such as acoustic signal analysis (36, 37) and AI-based predictive modeling (38).

This thematic shift is not solely driven by artificial intelligence itself, but rather by the growing integration of AI into existing medical frameworks, enhancing data interpretation, risk stratification, and clinical decision-making. It reflects a methodological evolution: from unidisciplinary problem-solving toward multidisciplinary and eventually interdisciplinary frameworks. In the backdrop of a new round of scientific and industrial revolutions, scientific research is going through a paradigm shift, making interdisciplinarity a necessity for disciplinary development in the era of big science, and this evolution enables more robust exploration of complex, multifactorial conditions like dysphagia, which involves neuromuscular, structural, and behavioral components (39). The growing prominence of ML and DL as keywords in recent years further supports the notion that computational modeling is becoming central to the study of swallowing pathophysiology.

Notably, the role of AI varies depending on the specific clinical situation. For instance, Radiotherapy vs. transoral robotic surgery and neck dissection for oropharyngeal squamous cell carcinoma (ORATOR): an open-label, phase 2, randomized trial exemplifies both the broad and narrow scopes of AI application in dysphagia research. From a broad perspective, robotic surgery can be considered part of AI's extended definition, reflecting the increasing role of intelligent systems in surgical interventions (40–42). From a narrower clinical standpoint, AI does not directly replace management of surgically treated diseases but serves an auxiliary role, supporting postoperative rehabilitation, risk assessment, and follow-up monitoring (43, 44).

## 6 Interdisciplinary gaps and collaborative opportunities

Interdisciplinarity, which addresses the complexity innate to nature and society, is the signature of complexity science (39). Despite notable advances in AI for dysphagia, the field faces significant challenges in achieving true interdisciplinary integration. Engineering and computer science currently dominate the authorship landscape, as exemplified by Sejdic Ervin, the most prolific author with 28 (4.42%) publications—while key clinical specialties such as speech-language pathology, rehabilitation, geriatric medicine and nursing remain underrepresented. Additionally, the concentration of high-impact studies in journals such as *Head and Neck*, and institutions like the University of Pittsburgh underscores the role of specialized academic hubs in shaping the discourse. However, countries' collaboration world map reveals a lack of meaningful collaboration: the USA ranks first among countries worldwide with 255 (40.28%) publications, accounting for almost half of the total. The top three institutions in terms of publication rankings are all from the USA, with very few from Asian countries and none from Africa and South America.

This lack of integration may slow the translational pipeline from algorithm development to clinical application, and exacerbate the problem of unequal distribution of global medical resources, especially in the field of swallowing disorders. Previous evidence has shown that interdisciplinary videoconferencing, as opposed to siloed consultation, can significantly reduce patient length of stay, streamline decision-making, and improve care efficiency (45). Interdisciplinarity is essential for disciplines to achieve development and solve problems, and is also valuable in breaking down disciplinary silos, enriching respective disciplines, achieving sustainable development, and producing multitalented dysphagia professionals (46), including those from gastroenterology, neurology, otolaryngology (ENT), speech-language pathology (SLP), nursing sci and clinical nutrition. Future efforts should thus prioritize cross-disciplinary, cross-institutional and cross-national collaboration, particularly between fields such as AI, medicine, nursing, and rehabilitation science.

## 7 Clinical and ethical significance

The prominence of the keyword dysphagia (286 occurrences), and its frequent co-occurrence with terms related to etiology and complications, suggests that AI holds promise not only in classification but also in mechanistic understanding and early risk detection. However, the paucity of highly cited studies addressing the clinical utility, ethical oversight, and real-world deployment of AI systems raises significant concerns (47, 48). While retrospective studies have demonstrated high diagnostic accuracy, the external validity and generalizability of these models, especially in older adults and neurodiverse populations—remain largely untested (49).

Moreover, ethical and legal implications, including data privacy, model transparency, and the potential for algorithmic bias, have yet to be adequately addressed (50). As AI systems begin to influence clinical decision-making, it is imperative that ethical frameworks evolve in parallel with technical advancements. Ensuring explainability, equity, and patient autonomy will be essential for the responsible and sustainable integration of AI into dysphagia care.

## 8 Limitations

This study has several limitations. First, only the Web of Science Core Collection was used, potentially omitting relevant studies indexed in other databases such as Scopus or PubMed. Second, non-English publications were excluded, possibly biasing the global landscape of AI in dysphagia. Third, citation-based metrics may not fully reflect the quality or impact of recent studies due to time-lag effects. Lastly, bibliometric tools may oversimplify complex interdisciplinary relationships, and this study did not assess algorithm performance or clinical applicability directly.

## 9 Conclusion

This study reveals the rapid growth and shifting research paradigm of AI in dysphagia, evolving from surgical interventions to intelligent, non-invasive diagnostics and rehabilitation. The United States and institutions like the University of Pittsburgh lead in output, yet international and interdisciplinary collaborations remain limited. Emerging focus on machine learning and deep learning signals a data-driven future, though clinical validation and ethical considerations remain underexplored. Moving forward, stronger cross-disciplinary collaboration is essential to translate AI innovations into effective and equitable dysphagia care.

## Data Availability

The datasets presented in this study can be found in online repositories. The names of the repository/repositories and accession number(s) can be found in the article/supplementary material.

## References

[1] Clavé P Shaker R. Dysphagia: current reality and scope of the problem. Nat Rev Gastroenterol Hepatol. (2015) 12:259–70. 10.1038/nrgastro.2015.49 25850008

[2] Rajati F Ahmadi N Naghibzadeh ZA Kazeminia M. The global prevalence of oropharyngeal dysphagia in different populations: a systematic review and meta-analysis. J Transl Med. (2022) 20:175. 10.1186/s12967-022-03380-0 35410274 PMC9003990

[3] Baijens LW Clavé P Cras P Ekberg O Forster A Kolb G . European society for swallowing disorders – European union geriatric medicine society white paper: oropharyngeal dysphagia as a geriatric syndrome. Clin Interv Aging. (2016) 11:1403–28. 10.2147/CIA.S107750 27785002 PMC5063605

[4] Ribeiro M Miquilussi PA Gonçalves FM Taveira KVM Stechman-Neto J Nascimento WV . The prevalence of oropharyngeal dysphagia in adults: a systematic review and meta-analysis. Dysphagia. (2024) 39:163–76. 10.1007/s00455-023-10608-8 37610669

[5] Mascarenhas A Mendo R O'Neill C Franco AR Mendes R Simao I . Current approach to dysphagia: a review focusing on esophageal motility disorders and their treatment. GE Port J Gastroenterol. (2023) 30:403–13. 10.1159/000529428 38476159 PMC10928869

[6] Labeit B Kremer A Muhle P Claus I Warnecke T Dziewas R . Costs of post-stroke dysphagia during acute hospitalization from a health-insurance perspective. Eur Stroke J. (2023) 8:361–9. 10.1177/23969873221147740 37021194 PMC10069210

[7] Bordeje Laguna L Marcos-Neira P de Lagran Zurbano IM Mor Marco E Pollan Guisasola C Vinas Soria CD . Dysphagia and mechanical ventilation in SARS-CoV-2 pneumonia: it's real. Clin Nutr. (2022) 41:2927–33. 10.1016/j.clnu.2021.11.018 34879968 PMC8608682

[8] Cosentino G Todisco M Giudice C Tassorelli C Alfonsi E. Assessment and treatment of neurogenic dysphagia in stroke and Parkinson's disease. Curr Opin Neurol. (2022) 35:741–52. 10.1097/WCO.0000000000001117 36226719

[9] Audag N Goubau C Toussaint M Reychler G. Screening and evaluation tools of dysphagia in adults with neuromuscular diseases: a systematic review. Ther Adv Chronic Dis. (2019) 10:2040622318821622. 10.1177/2040622318821622 30728931 PMC6357297

[10] Ferrari de Castro MA Dedivitis RA Luongo de Matos L Baraúna JC Kowalski LP de Carvalho Moura K . Endoscopic and videofluoroscopic evaluations of swallowing for dysphagia: a systematic review. Braz J Otorhinolaryngol. (2025) 91:101598. 10.1016/j.bjorl.2025.101598 40209342 PMC12013387

[11] Speyer R Baijens L Heijnen M Zwijnenberg I. Effects of therapy in oropharyngeal dysphagia by speech and language therapists: a systematic review. Dysphagia. (2010) 25:40–65. 10.1007/s00455-009-9239-7 19760458 PMC2846331

[12] Wheeler-Hegland K Ashford J Frymark T McCabe D Mullen R Musson N . Evidence-based systematic review: oropharyngeal dysphagia behavioral treatments. Part II–impact of dysphagia treatment on normal swallow function. J Rehabil Res Dev. (2009) 46:185–94. 10.1682/JRRD.2008.08.0094 19533532

[13] Cichero JAY Steele C Duivestein J Clavé P Chen J Kayashita J . The need for international terminology and definitions for texture-modified foods and thickened liquids used in dysphagia management: foundations of a global initiative. Curr Phys Med Rehabil Rep. (2013) 1:280–91. 10.1007/s40141-013-0024-z 24392282 PMC3873065

[14] de Lama Lazzara G Lazarus C Logemann JA. Impact of thermal stimulation on the triggering of the swallowing reflex. Dysphagia. (1986) 1:73–7. 10.1007/BF02407117

[15] Logemann JA Pauloski BR Colangelo L Lazarus C Fujiu M Kahrilas PJ. Effects of a sour bolus on oropharyngeal swallowing measures in patients with neurogenic dysphagia. J Speech Hear Res. (1995) 38:556–63. 10.1044/jshr.3803.556 7674647

[16] Wirth R Dziewas R Beck AM Clave P Hamdy S Heppner HJ . Oropharyngeal dysphagia in older persons - from pathophysiology to adequate intervention: a review and summary of an international expert meeting. Clin Interv Aging. (2016) 11:189–208. 10.2147/CIA.S97481 26966356 PMC4770066

[17] Shigematsu T Fujishima I Ohno K. Transcranial direct current stimulation improves swallowing function in stroke patients. Neurorehabil Neural Repair. (2013) 27:363–9. 10.1177/1545968312474116 23392916

[18] Jayasekeran V Singh S Tyrrell P Michou E Jefferson S Mistry S . Adjunctive functional pharyngeal electrical stimulation reverses swallowing disability after brain lesions. Gastroenterology. (2010) 138:1737–46. 10.1053/j.gastro.2010.01.052 20138037

[19] Ibrahim M Khalil YA Amirrajab S Sun C Breeuwer M Pluim J . Generative AI for synthetic data across multiple medical modalities: a systematic review of recent developments and challenges. Comput Biol Med. (2025) 189:109834. 10.1016/j.compbiomed.2025.109834 40023073

[20] Liu X Rivera SC Moher D Calvert MJ Denniston AK. Reporting guidelines for clinical trial reports for interventions involving artificial intelligence: the CONSORT-AI extension. Lancet Digit Health. (2020) 2:E537–48. 10.1136/bmj.m3164 33328048 PMC8183333

[21] Liu S Wright AP Patterson BL Wanderer JP Turer RW Nelson SD . Using AI-generated suggestions from ChatGPT to optimize clinical decision support. J Am Med Inform Assoc. (2023) 30:1237–45. 10.1093/jamia/ocad072 37087108 PMC10280357

[22] Lee SJ Ko JY Kim HI Choi SI. Automatic detection of airway invasion from videofluoroscopy via deep learning technology. Appl Sci. (2020) 10:6179. 10.3390/app10186179

[23] Li D Zhang Y Wu J Luo W Liu T Lyu B . Development of the swallowing activity classification model for old adults based on acoustic analysis. Innov Aging. (2024) 8(Supplement_1):927. 10.1093/geroni/igae098.2991

[24] Donthu N Kumar S Mukherjee D Pandey N Lim WM. How to conduct a bibliometric analysis: an overview and guidelines. J Bus Res. (2021) 133:285–96. 10.1016/j.jbusres.2021.04.070

[25] Zhu J Liu W. A tale of two databases: the use of web of science and scopus in academic papers. Scientometrics. (2020) 123:321–35. 10.1007/s11192-020-03387-8

[26] Shen Z Wu H Chen Z Hu J Pan J Kong J . The global research of artificial intelligence on prostate cancer: a 22-year bibliometric analysis. Front Oncol. (2022) 12:843735. 10.3389/fonc.2022.843735 35299747 PMC8921533

[27] Sun W Kang X Zhao N Dong X Li S Zhang G . Study on dysphagia from 2012 to 2021: a bibliometric analysis via CiteSpace. Front Neurol. (2022) 13:1015546. 10.3389/fneur.2022.1015546 36588913 PMC9797971

[28] LaPolla FWZ. Excel for data visualization in academic health sciences libraries: a qualitative case study. J Med Libr Assoc JMLA. (2020) 108:67–75. 10.5195/jmla.2020.749 31897053 PMC6919992

[29] Pan X Yan E Cui M Hua W. Examining the usage, citation, and diffusion patterns of bibliometric mapping software: a comparative study of three tools. J Informetr. (2018) 12:481–93. 10.1016/j.joi.2018.03.005

[30] Aria M Cuccurullo C. Bibliometrix: an R-tool for comprehensive science mapping analysis. J Informetr. (2017) 11:959–75. 10.1016/j.joi.2017.08.007

[31] Nichols AC Theurer J Prisman E Read N Berthelet E Tran E . Radiotherapy versus transoral robotic surgery and neck dissection for oropharyngeal squamous cell carcinoma (ORATOR): an open-label, phase 2, randomised trial. Lancet Oncol. (2019) 20:1349–59. 10.1016/S1470-2045(19)30410-3 31416685

[32] Janiesch C Zschech P Heinrich K. Machine learning and deep learning. Electron Mark. (2021) 31:685–95. 10.1007/s12525-021-00475-2

[33] Choi RY Coyner AS Kalpathy-Cramer J Chiang MF Campbell JP. Introduction to machine learning, neural networks, and deep learning. Transl Vis Sci Technol. (2020) 9:14. 10.1167/tvst.9.2.14 32704420 PMC7347027

[34] Castiglioni I Rundo L Codari M Di Leo G Salvatore C Interlenghi M . AI applications to medical images: from machine learning to deep learning. Phys Med. (2021) 83:9–24. 10.1016/j.ejmp.2021.02.006 33662856

[35] Iseli TA Kulbersh BD Iseli CE Carroll WR Rosenthal EL Magnuson JS. Functional outcomes after transoral robotic surgery for head and neck cancer. Otolaryngol Head Neck Surg. (2009) 141:166–71. 10.1016/j.otohns.2009.05.014 19643246

[36] Li D Wu J Jin X Li Y Tong B Zeng W . A review on intelligent aid diagnosis for dysphagia using swallowing sounds. Interdiscip Nurs Res. (2023) 2:250–6. 10.1097/NR9.0000000000000040

[37] Kim H Park HY Park D Im S Lee S. Non-invasive way to diagnose dysphagia by training deep learning model with voice spectrograms. Biomed Signal Process Control. (2023) 86:105259. 10.1016/j.bspc.2023.105259

[38] Lienhart AM Kramer D Jauk S Gugatschka M Leodolter W Schlegl T. Multivariable risk prediction of dysphagia in hospitalized patients using machine learning. Stud Health Technol Inform. (2020) 271:31–8. 10.3233/SHTI200071 32578538

[39] Qiao J. Interdisciplinarity for life sciences. Interdiscip Nurs Res. (2022) 1:1. 10.1097/NR9.0000000000000005

[40] Chatterjee S Das S Ganguly K Mandal D. Advancements in robotic surgery: innovations, challenges and future prospects. J Robot Surg. (2024) 18:28. 10.1007/s11701-023-01801-w 38231455

[41] Knudsen JE Ghaffar U Ma R Hung AJ. Clinical applications of artificial intelligence in robotic surgery. J Robot Surg. (2024) 18:102. 10.1007/s11701-024-01867-0 38427094 PMC10907451

[42] Haidegger T Speidel S Stoyanov D Satava RM. Robot-assisted minimally invasive surgery-surgical robotics in the data age. Proc IEEE. (2022) 110:835–46. 10.1109/JPROC.2022.3180350

[43] Jae LS. Application of artificial intelligence in the area of dysphagia. J Korean Dysphagia Soc. (2020) 10:4–9. 10.34160/jkds.2020.10.1.002

[44] Alter IL Dias C Briano J Rameau A. Digital health technologies in swallowing care from screening to rehabilitation: a narrative review. Auris Nasus Larynx. (2025) 52:319–26. 10.1016/j.anl.2025.05.002 40403345 PMC12354047

[45] Reeves S Pelone F Harrison R Goldman J Zwarenstein M. Interprofessional collaboration to improve professional practice and healthcare outcomes. Cochrane Database Syst Rev. (2017) 6:CD000072. 10.1002/14651858.CD000072.pub3 28639262 PMC6481564

[46] Shang S Li D Hou L Yu M Wang Z. Interdisciplinarity catalyzes sustained development of nursing discipline. Interdiscip Nurs Res. (2022) 1:3–5. 10.1097/NR9.0000000000000009

[47] Roldan-Vasco S Restrepo-Uribe JP Orozco-Duque A Suarez-Escudero JC Orozco-Arroyave JR. Analysis of electrophysiological and mechanical dimensions of swallowing by non-invasive biosignals. Biomed Signal Process Control. (2023) 82:104533. 10.1016/j.bspc.2022.104533

[48] Girardi AM Cardell EA Bird SP. Artificial intelligence in the interpretation of videofluoroscopic swallow studies: implications and advances for speech-language pathologists. Big Data Cogn Comput. (2023) 7:178. 10.3390/bdcc704017839961823

[49] Ashok M Madan R Joha A Sivarajah U. Ethical framework for artificial intelligence and digital technologies. Int J Inf Manag. (2022) 62:102433. 10.1016/j.ijinfomgt.2021.102433

[50] Cartolovni A Tomičić A Lazić Mosler E. Ethical, legal, and social considerations of AI-based medical decision-support tools: a scoping review. Int J Med Inf. (2022) 161:104738. 10.1016/j.ijmedinf.2022.104738 35299098

